# Facilitators and barriers to behaviour change within a lifestyle program for women with obesity to prevent excess gestational weight gain: a mixed methods evaluation

**DOI:** 10.1186/s12884-021-04034-7

**Published:** 2021-08-18

**Authors:** Rebecca F. Goldstein, Jacqueline A. Boyle, Clement Lo, Helena J. Teede, Cheryce L. Harrison

**Affiliations:** 1 Monash Centre for Health Research and Implementation, School of Public Health and Preventive Medicine, Monash University, Level 1, 43-51 Kanooka Gve, Clayton, 3168 Australia; 2 Diabetes and Vascular Medicine Unit, Monash Health, Clayton, 3168 Australia; 3 Monash Women’s, Monash Health, Clayton, 3168 Australia

**Keywords:** Gestational weight gain, Obesity, Health coach, Intervention, Implementation, Qualitative, Health professionals, Pregnant women, Mixed methods

## Abstract

**Background:**

Maternal obesity is associated with health risks for women and their babies and is exacerbated by excess gestational weight gain. The aim of this study was to describe women’s experiences and perspectives in attending a Healthy Pregnancy Service designed to optimise healthy lifestyle and support recommended gestational weight gain for women with obesity.

**Methods:**

An explanatory sequential mixed methods study design utilised two questionnaires (completed in early and late pregnancy) to quantify feelings, motivation and satisfaction with the service, followed by semi-structured interviews that explored barriers and enablers of behaviour change. Data were analysed separately and then interpreted together.

**Results:**

Overall, 49 women attending the service completed either questionnaire 1, 2 or both and were included in the analysis. Fourteen women were interviewed. Prior to pregnancy, many women had gained weight and attempted to lose weight independently, and reported they were highly motivated to achieve a healthy lifestyle. During pregnancy, diet changes were reported as easier to make and sustain than exercise changes. Satisfaction with the service was high. Key factors identified in qualitative analysis were: service support enabled change; motivation to change behaviour, social support, barriers to making change (intrinsic, extrinsic and clinic-related), post-partum lifestyle and needs. On integration of data, qualitative and quantitative findings aligned.

**Conclusions:**

The Healthy Pregnancy service was valued by women. Barriers and enablers to the delivery of an integrated model of maternity care that supported healthy lifestyle and recommended gestational weight gain were identified. These findings have informed and improved implementation and further scale up of this successful service model, integrating healthy lifestyle into routine antenatal care of women with obesity.

**Trial registration:**

This trial is registered with the Australian New Zealand Clinical Trials Registry (no.12620000985987). Registration date 30/09/2020, retrospectively registered. http://www.anzctr.org.au/

**Supplementary Information:**

The online version contains supplementary material available at 10.1186/s12884-021-04034-7.

## Contributions to literature

- Randomised controlled trials designed to improve healthy lifestyle, limit gestational weight gain and improve maternal and infant outcomes are effective. The priority and remaining challenge is to implement these programs into usual maternal care
- Successful programs require dedicated, well-trained health professionals that can educate, empower and support women to make changes
- This mixed-methods study identifies barriers and enablers to the delivery of an integrated model of maternity care. These findings contribute to gaps in the literature and will inform and improve implementation and further scale-up of this successful service model

## Background

Maternal obesity and excessive gestational weight gain (GWG) both independently contribute to adverse maternal and neonatal outcomes [1, 2], as well as to increased risk of postpartum obesity development in mothers and their children [3, 4]. The National Academy of Medicine (previously Institute of Medicine, IOM) recommendations for healthy GWG are specific to a woman’s pre-pregnancy BMI [5]. A systematic review and meta-analysis [6] of more than one million women demonstrated that almost half gained above GWG recommendations leading to adverse maternal and neonatal outcomes. Women above a healthy weight preconception had the highest prevalence of excess GWG [7].

Pregnancy has long been considered ‘a teachable moment’ for optimised weight gain and obesity prevention [8]. Interventions designed to improve healthy lifestyle, limit gestational weight gain and improve maternal and infant outcomes are effective, supported by level I evidence from a large meta-analysis [9]. The priority and remaining challenge is to implement these programs used in randomised controlled trials (RCTs) into usual maternal care [10, 11]. Successful programs require dedicated, well-trained health professionals that can educate, empower and support women to make changes [12]. Barriers include antenatal health professionals lack of skills, time and confidence discussing sensitive issues [13–15], and women can feel stigmatised if staff are not well trained [16]. Generally, women are motivated to make positive changes, but want support and direction from qualified health professionals (e.g. midwives, dieticians, allied health professionals) [17, 18].

The Healthy Pregnancy service was established in 2015 at Monash Health, the largest health service in Australia, to care for women with a pre-pregnancy BMI of ≥35 kg/m^2^. This co-designed antenatal service integrates an embedded, evidence-based lifestyle intervention, the HeLP-her Healthy Lifestyle in Pregnancy Intervention [19]. The HeLP-her program intervention has been shown to be effective in reproductive aged women in multiple settings outside of pregnancy as well as in pregnancy, including women at increased risk of gestational diabetes in routine antenatal care [20–23] and is also cost effective [24]. The program is underpinned theoretically by The Social Cognitive Theory and promotes goal setting, self-monitoring, social support and problem solving [22]. Here, in women with obesity at high risk of pregnancy complications, dedicated staff including a physician (endocrinologist) and health coach delivered the program, designed to address the barriers of time constraints and expertise of routine maternity health professionals. The health coach is a qualified exercise physiologist with qualifications in behavioural neuroscience and expertise in motivational interviewing in reproductive aged women for weight gain prevention. This model of care project was designed to incorporate pragmatic implementation research, where evidence is generated in the context of usual clinical care [13, 25, 26].

Mixed methods approaches in implementation research apply both quantitative and qualitative approaches to provide novel insights on implementation of models of care [27]. Here, the aim of this study was to apply mixed methods to explore the experiences and perspectives of the women attending the integrated antenatal clinic and Healthy Pregnancy service, to understand the barriers and enablers to lifestyle change and to identify how this service can be improved to inform sustainable implementation and scale-up.

## Methods

### Study design

An explanatory sequential mixed methods study design was used [27]. This two-phase design involved using two questionnaires completed by pregnant women at different time points during pregnancy, followed by qualitative interviews. Using this approach, the qualitative results aimed to expand and confirm findings from the quantitative phase and data was integratedin analysis. The consolidated criteria for reporting qualitative research (COREQ) 32-item checklist was used in planning and reporting [28]. This study was approved by the Monash Health Ethics committee (RES-17-0000-313 L).

### Service setting

This study is part of a broader pragmatic implementation trial (The Healthy Lifestyle in Pregnancy Project, HiPP) that evaluated the effect of a lifestyle intervention on gestational weight gain and maternal and infant outcomes in women with maternal obesity (Australian New Zealand Clinical Trials Registry: 12620000985987). The project was implemented within a large hospital network in metropolitan Melbourne, Australia, with approximately 10,000 live births per year. Australia offers universal freely accessible healthcare and Monash Health is the largest health service nationally, situated in a catchment with a low socio-economic status (SES), diverse ethnic background population [29]. The specific service provided care to women with a BMI of 35–43 kg/m^2^ with approximately 200 live births per year. Women with a BMI of > 43 kg/m^2^ were triaged to deliver at the tertiary hospital site to accommodate their additional needs as standard approach within the health service.

The Healthy Pregnancy service embedded a patient-led behaviour change lifestyle intervention delivered by a health Coach and a physician (intervention staff) over five sessions integrated with routine pregnancy care. The physician also managed medical complications in pregnancy including gestational diabetes. The intervention was largely delivered by the health coach and focused on behaviour change and self-management of weight, healthy diet and exercise. Skills were practised in goal setting, problem solving and relapse prevention, with the aim to achieve small, sustainable changes to healthy lifestyle behaviours. The sessions were one-on-one and between 20 and 40 min in duration. The first session was longer, with both education provision (i.e. dietary and physical activity guidelines for pregnancy, GWG recommendations) and behavioural skills discussed and practiced. The study design was cognisant of the clinical demands of midwives and obstetricians who are generally time-poor and lack the training and confidence to spend prolonged periods counselling women on healthy lifestyle [30]. Therefore, midwives and obstetricians in the Healthy Pregnancy service did not deliver the behaviour change intervention, but were part of the team and were supportive of the program messages and reinforced this with women throughout.

Women who attended the Healthy Pregnancy service between 2016 and 2018 were compared to standard care (those not receiving embedded lifestyle intervention) for the primary outcome of GWG and secondary outcome of maternal and infant outcomes and implementation knowledge [31]. Detailed study design is described previously: the first intervention session coincided with the first medical review, typically between 12 and 18 weeks, and final session at ~ 36 weeks. Intervention uptake was 95, and 87% of women attended 80% or more of the 5 sessions. Health professionals’ perspectives of the service also studied [12].

### Questionnaire design

Questionnaires were developed to understand pregnant women’s experience in attending the service to identify barriers and enablers to behaviour change. They were developed by a team with multidisciplinary expertise, including dietetics, exercise physiology, obstetrics, endocrinology and psychology. While the questionnaires were not piloted here, they were based on priori questionnaires used in over a decade of research focused on obesity prevention in reproductive aged women. These include observational studies evaluating health related behaviours among women with gestational diabetes mellitus [32], polycystic ovary syndrome and type 2 diabetes [33] as well as RCTs to limit gestational weight gain [22, 23, 34] and prevent weight gain in non-pregnant women [19, 20] (studies performed by our group). Here, we used the validated self-management questionnaire for health-related diet and exercise behavioursby Sallis [35] . To our knowledge, there is no standard or validated approach to evaluating aspects of care evaluated in our study, which therefore informed the use of our internally developed questionnaires here.

Questionnaire one was completed at the first (or close to) the initial session (12–18 weeks). Questions assessed demographics, basic diet and physical activity, risk perception, motivation and readiness to change and self-management strategies. Questionnaire two was completed at (or just after) the final session (36 weeks). Questions assessed satisfaction with service, changes made in pregnancy and corresponding barriers, and self-management strategies.

Some questions included a statement (e.g. I think it is important to have a healthy lifestyle during pregnancy) with responses on a 5-point Likert scale. In keeping with a pragmatic clinic trial approach, we did not intend for all women at the Healthy Pregnancy Service to complete the questionnaires, but rather a percentage of these. Questionnaires were initially distributed by mail in early 2017; between April 2017 and February 2018, the questionnaires were distributed in person by a researcher/clinician at the Healthy Pregnancy Service and completed in the clinic. Questionnaire 1 and 2 are in the Additional files 1 and 2.

### Qualitative interviews

Semi-structured interviews (Additional file 3) were conducted with a sample of women attending clinic to gain a deeper understanding of women’s experience attending the service, and the barriers and enablers to behaviour change. Purposive sampling targeted women who were more than 31–32 weeks gestation, were representative of parous and nulliparous as well as those with and without GDM, and would have attended a substantial proportion of their intervention care. Women participating in the qualitative analysis could do so without participating in the quantitative component. Questions were developed based on a preliminary analysis of the questionnaires. Participants were recruited by RG (a female clinician-researcher) in person. Interviews were conducted over the phone by RG, who had postgraduate expertise in qualitative methods. RG worked in the clinic as a physician for 1 year prior to commencement of the research project and then ceased clinical work to focus on the research. Written informed consent was obtained from all participants prior to the interview. The interviews were between 10 and 25 min duration and conducted in July and August 2017. Data from the interviews was audiotaped and transcribed verbatim by an independent transcribing service. Participant details were deidentified for anonymity. Interviews were collected until data saturation was reached, determined when no new ideas emerged from the interviews.

### Data analyses

#### Quantitative data

Analysed using STATA software, version 15.0. Categorical data were presented as frequency and percentage (n (%)). Continuous data were presented as mean (standard deviation). Responses to 5-point Likert-scaled questions (e.g. strongly agree, agree, somewhat agree, disagree, strongly disagree; or daily, weekly, monthly, occasionally, never; or always, very often, often, occasionally, never) were collapsed into 2 categories (agree/disagree; or regularly/rarely; or often/rarely) respectively. Mann Whitney test (Wilcoxon signed-rank) was used to compare responses to questions repeated in questionnaire 1 and 2.

#### Qualitative data

Transcripts were independently analysed and coded by two researchers (RG and CL) using the NVivo 12 software (QSR International Pty Ltd. 2018). Data was searched for concepts in relation to interview questions. Codes were grouped into themes using inductive analysis to meet the aims of the study, in a constant comparative manner using a generic approach as described by Patton [36] and Harding [37]. The objective was for the themes to be strongly linked to the data, so an inductive approach was chosen using raw data to derive the structure of analysis. Consensus regarding the emerging themes was reached between the two researchers.

Integration of qualitative and quantitative data was performed with a mixed methods design using the approach described by Fetters et al. design [27]. At the study design level, an explanatory sequential design was used; the quantitative data was collected and analysed first, informing the qualitative data collection and analysis. At the interpretation and reporting level, integration occurred through narrative, with a contiguous approach.

## Results

### Quantitative phase

Questionnaires were initially distributed by mail in early 2017, however the response rate was poor (14%). Between April 2017 and February 2018, the questionnaires were distributed in person and completed in the clinic (96% response rate).

Of the 157 women attending the lifestyle intervention between 2016 and 2018, 58 women completed either questionnaire 1, 2 or both. After excluding 9 women (either did not attend lifestyle intervention clinic, BMI outside of range, moved to another health service), 49 women were included in the analysis: 44 completed questionnaire 1, 40 completed questionnaire 2 and 35 completed both questionnaire 1 and 2. The demographics of questionnaire 1 participants are shown in Table 1.

**Table 1:** Demographics of questionnaire 1 participants (*n* = 44)

Demographic	mean (SD) or no. (%)
**Age (years)**	29.6 (4.7)
**BMI (kg/m**^**2**^**)**	38.5 (2.1)
**Parity**	
0	22 (50)
1	12 (27)
2	7 (16)
3	2 (5)
4	1 (2)
**Highest level of schooling**	
year 10/11	9 (20)
year 12	10 (22)
post school certificate/diploma	15 (34)
bachelor degree and above	10 (22)
**Employment**	
full time	16 (36)
Part time/casual	15 (34)
no paid work	13 (30)
**Average yearly income**	
< $40,000	10 (24)
41–64,000	11 (27)
65–80,000	7 (17)
> 81,000	15 (33)
**GDM**	
yes	15 (34)
no	29 (66)
**total GWG (kg)**	6.46 (4.4)

#### Questionnaire 1

##### Weight and lifestyle

In early pregnancy, 21 (48%) of women reported weighing themselves regularly and 23 (52%) rarely. Thirty-one (70%) had gained weight in the past year before pregnancy: 9 (28%) ≤4 kg, 6 (19%) 5 kg, 14 (44%) 6-10 kg and 3 (9%) > 10 kg.

Prior to pregnancy, 38 (80%) had attempted weight loss in the last year, and 14 (32%) had consulted a health professional to manage their weight. To improve their lifestyle, 17 (39%) reported increasing vigorous exercise, 33 (75%) reduced portion size and 33 (75%) reduced snack foods/takeaway.

Before pregnancy, 38 (86%) were dissatisfied with their weight and 29 (89%) were dissatisfied with their body shape.

##### Risk perceptions, health beliefs, stage of change

Twenty-seven (63%) of women identified that 5-9 kg is the ideal weight gain in pregnancy (consistent with guideline recommendations), 14 (33%) thought 0-5 kg was appropriate. Twenty-six (65%) recognised that increased weight gain was not associated with more nutrients for the baby, whereas increased weight was reported as associated with big babies/macrosomia (17 (41%)), diabetes in pregnancy/gestational diabetes (25 (61%)) and high blood pressure in pregnancy (14 (34%)).

Early in pregnancy, 42 (95%) agreed that a healthy lifestyle in pregnancy is important and 37 (84%) thought they were at risk of excess weight gain, whilst 43 (100%) believed they could manage healthy lifestyle and weight gain in pregnancy, and 43 (97%) intended to take actions to prevent excess weight gain.

##### Readiness to change

**Fig. 1:**
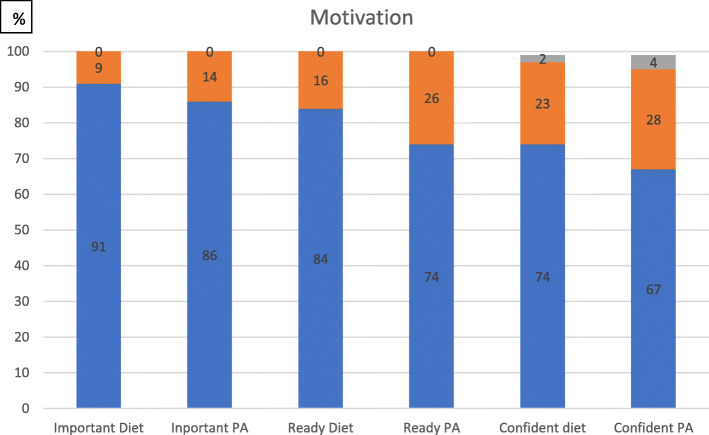
Motivation

Motivation was assessed in Fig. 1. Participants rated importance/readiness/confidence in making healthy lifestyle changes during pregnancy regarding diet/physical activity (PA), and responses were on a scale of 0–10 (0 not at all, to completely 10).

#### Questionnaire 2

##### Satisfaction with the healthy pregnancy service

**Fig. 2:**
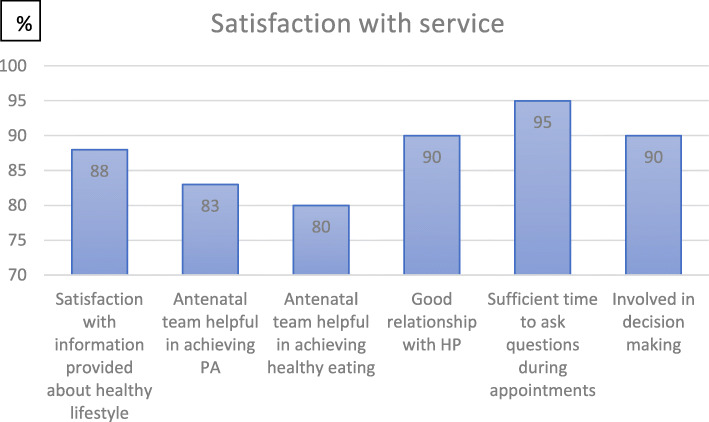
Satisfaction with service

This is described in Fig. 2. Participants rated their satisfaction with information provided by the antenatal team and their relationship with the health professionals.

##### Changes made during pregnancy

Overall, 34 (87%) of women reported taking actions during pregnancy to achieve a healthy lifestyle, with the main changes being increasing water (26 (66%)) and fruit and vegetables (23 (59%)), and reducing takeaway (20 (51%)). Less women made changes to physical activity, with increased number of exercise sessions (14 (36%)) and increased time on sessions (3(8%)).

##### Maintaining change

Overall, 24 (65%) worked on maintaining changes to diet and 19 (51%) worked on maintaining changes to physical activity, whilst 28 (74%) were confident they could maintain lifestyle changes post-partum.

##### Barriers to lifestyle change

Women reported fatigue as the main barriers to lifestyle change (32 (82%)), followed by lack of time (18 (46%)), taking care of other children (14 (36%)) and no motivation (10 (26%)).

#### Comparison of questionnaire 1 and 2

Selected responses to a 29 question survey of self-management strategies based on an adapted tool by Sallis [35] completed early and late in pregnancy are compared in Table 2.

**Table 2:** Self-management strategies

	Frequency	Questionnaire 1 n (%)	Questionnaire 2 n (%)	Comparison Mann- Whitney (***p***-value)
**PHYSICAL ACTIVITY (PA)**				
Think about benefits from PA	Often	33 (75)	32 (80)	0.53
I know when I should do more	Often	36 (82)	31 (79)	1.00
Plan ahead of time	Often	35 (57)	23 (58)	0.71
Stick to my plans	Often	26 (59)	23 (58)	0.52
Make backup plans to get PA	Often	14 (32)	11 (28)	1.00
Keep track of PA	Often	18 (41)	18 (46)	0.53
Able to get back on track when off-track	Often	19 (43)	21 (53)	0.21
**DIET**				
Watch what I eat	Often	32 (74)	30 (77)	0.74
Keep track of diet	Often	24 (55)	26 (67)	0.10
If don’t eat well, think about ways to improve	Often	27 (61)	28 (72)	0.16
Make time to prepare healthy meals	Often	19 (43)	27 (69)	0.004
Have quick healthy food available	Often	22 (51)	29 (76)	0.01
Try new healthy foods and recipes	Often	22 (52)	30 (74)	0.02
Eat healthy food	Often	30 (68)	31 (79)	0.10
Replace snacks with healthier choice	Often	24 (56)	29 (74)	0.008
**WEIGHT**				
I watch my weight	Often	27 (61)	27 (69)	0.21
I weigh myself regularly	Often	20 (45)	20 (51)	0.008

### Qualitative phase

Fourteen women agreed to participate in an interview and all were interviewed. Women were a mix of nulliparous and parous, with and without GDM, and most had completed some post school education and were employed. The demographics of interview participants are shown in Table 3.

**Table 3:** Demographics of interview participants (*n* = 14)

***Participant no***	***Age range***	***Gestation***	***Parity***	***GDM***	***Pre-pregnancy BMI (kg/m***^***2***^***)***	***total GWG (kg)***	***Highest level of schooling***	***Work***	***Income***
*1*	*20–24*	*36*	*0*	*no*	*36.9*	*7.3*	*post school certificate*	*full time*	*$65–80,000*
*2*	*35–40*	*33*	*1*	*no*	*39*	*9.7*	*post school certificate*	*part-time*	*$ > 81,000*
*3*	*20–24*	*34*	*0*	*no*	*38.2*	*9*	*year 10/11*	*no paid work*	*<$40,000*
*4*	*30–34*	*34*	*0*	*no*	*38.7*	*5.6*	*bachelor degree*	*full time*	*$ > 81,000*
*5*	*35–40*	*31*	*2*	*no*	*41.3*	*2.5*	*bachelor degree*	*part-time*	*$65–80,000*
*6*	*25–29*	*32*	*0*	*no*	*42*	*10.2*	*bachelor degree*	*full time*	*>$81,000*
*7*	*20–24*	*31*	*1*	*no*	*42*	*3.6*	*year 12*	*part-time*	*>$81,000*
*8*	*25–29*	*34*	*2*	*no*	*39.7*	*8.4*	*year 10/11*	*no paid work*	*$65–80,000*
*9*	*30–34*	*31*	*0*	*no*	*37.6*	*12.1*	*bachelor degree*	*part-time*	*$41–64,000*
*10*	*30–34*	*34*	*0*	*yes*	*35.4*	*−2.5*	*post school certificate*	*casual*	*>$81,000*
*11*	*35–40*	*32*	*0*	*yes*	*42*	*3.8*	*post school certificate*	*full time*	*>$81,000*
*12*	*30–34*	*37*	*2*	*yes*	*36*	*8*	*post school certificate*	*full time*	*>$81,000*
*13*	*25–29*	*36*	*1*	*yes*	*36.2*	*14.4*	*post school certificate*	*no paid work*	*$41–64,000*
*14*	*25–29*	*36*	*0*	*no*	*36.9*	*10.3*	*year 12*	*part-time*	*<$40,000*

Interview themes are summarised in Table 4 and described with example quotes below.

**Table 4:** Themes and subthemes

Themes and sub-themes	Barrier	Enabler
THEME 1. Service support enabled change		
Subthemes		
• Rapport with staff		+
• Advice provided		+
• Awareness created change		+
THEME 2. Drivers of motivation to behaviour change		
Subthemes		
• Women’s own motivation		+
• Potential health consequences		+
• Being accountable to healthcare professionals	+	+
THEME 3. Social support		+
THEME 4. Barriers to making change		
• Personal: intrinsic and extrinsic	+	
• Clinic-related	+	
THEME 5. Post-partum lifestyle and needs		
• Sustainable lifestyle changes	+	+
• Support required to maintain lifestyle changes		+

The + corresponds to the sub-themes (whether they were barriers or enablers)

#### THEME 1. Service support enabled change

Most women felt that the service and the intervention staff (health coach and physician) enabled positive behaviour changes. Key strengths included developing rapport, delivering clear advice and providing awareness that created change.

- Sub-theme: Rapport with women

Women described the intervention staff as interested in their well-being and acknowledged the comfortable environment created. Staff involved them in decision making and supported the decisions women made.

*“compared to other doctors, that they were really interested in what you have to say. They gave honest opinions, and feedbacks, um, which I really liked as well... I felt like very comfortable when I was talking to everyone, so, um, yeah, it was a very… it’s a very positive experience going through that clinic”. (participant #1)*

*“they would always asked me those questions. “Are you still going through those issues? Do you want to try and, um, find another… another solution? Um, do you want to try and take another approach?” … I was very pleased…they weren’t pushing or pressuring, or anything. So they weren’t persistent. Um, which is also good because you can’t really pressure someone into doing things” (participant #1)*

- Sub-theme: Advice provided

Women reported receiving clear information around diet, exercise and weighing goals that was easy to take on board and felt the level of support helped them make changes. The intervention team had realistic expectations of achievable goals and provided personalised advice.

*“I’ve obviously got restrictions on what I can do because I’ve got other health concerns but um they tried to work around that, you know, with things that I obviously do – can do, so like swimming and stuff like that. They were very encouraging about participating in those.” (participant #4)*

*“Exercise has been restricted for me, because I had a sub chorionic haemorrhage earlier on, and the blood clot still hasn’t absorbed. So I’ve been put on exercise restrictions, but they were really good with giving me exercises that I am allowed to do. Like walking, and um, the physician even suggested like sometimes while I’m sitting down watching TV, just to have like, light two kilo dumbbells, and just to be using them with my arms. Um, just to be like, burning a few excess calories without putting anything at risk. Um, so I found that really helpful.” (participant #7)*

Some women reported that the regular weighing at the clinic helped keep them motivated, particularly if they were not likely to weigh themselves at home.

*“I found it really helpful that she tracked my weight because I don’t do that very well. So I can see through her that, you know, my weight is increasing dramatically or if it’s slowing down… I like being able to see data and having that presented to me kind of changes my mind and changes my opinion a lot and from that I can then go and do things” (participant #9)*

- Sub-theme: Awareness created change and improved satisfaction

Women were able to identify changes that they made after planning with the intervention staff, and a number reported feeling more confident as a result of making changes.

*“I was more aware of what I was eating and portions. I find that when you buy food out as well, the portions are way too big…my biggest example just the other day was I got one of those boxes of noodles that you get…and it’s probably three servings. I didn’t realise that…but when I put it in a bowl out of the box – it changes everything” (participant #4*)

*“one of my cravings was ice cream and they told me to substitute it for frozen yogurt if I could. So I did that, and then the craving for ice cream kind of went away. So I cut that out as well. Also with like milk and cheese, they just said to choose the light option if I could, if I didn't mind the flavour and stuff like that. So I tried that as well and that was good.” (participant #3)*

One participant who was pregnant with her third child, reflected that the intervention gave her tools to limit gestational weight gain that would have been helpful in her earlier pregnancies.

*“I never had a health coach with my first or second, and I wish I did. So with my first… I put on 36 kilos. With my second I put on 19. I’m now on my third, and I’ve only put on seven. So I wish that back then, when I did have my daughter, and put on 36 kilos, that there was a health coach to show me the rights and wrongs.” (participant #7)*

#### THEME 2. Drivers of motivation to behaviour change

Women described different drivers to making behaviour change, depending on their personal experience.

- Subtheme: Women’s own motivation

Those with previous pregnancies or better health literacy came in with more experience and were able to implement changes more independently, sometimes initiating changes before attending the clinic.

*“I was very confident to start with because it’s not my first. So having already had two kids, this is sort of routine. I kind of know what to expect and what’s coming and all of that sort of thing. So yeah, I was fairly confident to start with. Um, seeing them each time and, you know, getting weighed and knowing that I’m definitely doing the right thing, that helped” (participant #5)*

*“knowing that I was pregnant and that I had gestational diabetes (previously), and the chances of me having it again was higher, so making sure that we push forward with changing our diet early on.” (participant #12)*

Some women described their motivation as being intrinsic, feeling that the responsibility of eating well lay within themselves.

*“all of these (changes) were up to me and it was up to me to make them work. I could gore down a whole pack of doughnuts if I wanted to but it was about controlling it. So I would be the one that would hold myself back if I slipped.” (participant 4)*

- Sub-theme: Potential health consequences for baby

Women were motivated to make changes, believing that this impacted the health of their child. These feelings were heightened if they developed a complication in pregnancy, with an awareness that lack of action could affect the baby. The fear of experiencing an adverse outcome acted as a significant motivator.

*“it was more that I was aware that it wasn’t just my own health that I was impacting, it was also the baby’s, so I had to do these things to give him the best possible chance.” (participant #4)*

*“when I found out I had the diabetes. I made really big changes... when it was, you know, for a reason, it was like, “Yeah, I really do need to change that. And I can swap that; it’s that easy. I can swap that.” (participant #8)*

*“at the start of my pregnancy like I came back…I did Down Syndrome test, and it came back as a high risk. And then that kind of you know - and I just wanted to take care of my baby throughout this pregnancy. Like it was, it was a lot different to my first one.” (participant #13)*

*“I was a little bit fearful, and that's why I found I had to make those changes.” (participant #11)*

- Sub-theme: Being accountable to healthcare professionals

Many women found the regular appointments with the intervention staff to check on their progress as a technique for keeping on track.

*“I did find the accountability very helpful, um, someone actually checking up on me and making sure I’m staying on track and I’m doing the right thing.” (participant #14)*

*“sometimes I would fall back into my old ways, and it would encourage me to you know like - she would like encourage me to do like better, and you know eat the proper foods, and exercise a bit more. Yeah. She was good”. (participant #13)*

In contrast, a small number of women described fear of disapproval from the staff for not meeting desired weight goals. This could be seen as a barrier for these women.

*“sometimes I was really nervous that I was going to go in there and they would be like, “Oh no, you’re putting on too much weight” or something like that and I was a little bit worried about that because I know that they weigh me and, you know, those are always really nervous times” (participant #4)*

#### THEME 3. Social support

A number of women were very open with their family about lifestyle change, and in return received moral support from their family, their partner in particular.

*“I had that support from my husband, because you know, he does come around with me on these walks, so he’s, um, a bit more encouraging with that. So you know, he said, “if you can’t do half an hour, we’ll walk for 20 minutes now, and then in an hour’s time, we’ll walk another 20 minutes or so.”” (participant #1)*

*“my mum’s been doing it with me, so that’s good…and my dad’s been really good with watching my daughter and stuff when we go out, and you know, if we can’t take her with us... Um, so yeah, I mean, they’ve been really supportive in the fact that I need to do the… you know, the extra exercise” (participant #7)*

In some circumstances, women’s changes had a positive flow on affect to other family members, with family making changes to the types of food purchased and participating in exercise.

*“my mother, um, she’s started buying things that, you know, that I …wouldn’t have eaten before. So she’ll have skinny milk in the fridge if I come over and things like that” (participant #4)*

*“And my husband’s been fantastic. He’s changed uh pretty much a lot of the things that he eats himself to support me. So we don’t have white bread in the house anymore. We have seeded bread and things like that. So um yeah, my family and friends have been fantastic. My husband is exercising much more now as well. Um, so he’s uh more aware of himself in general. It’s good.” (participant #4)*

*“my husband is very supportive of healthy eating. He wants us all to be healthy. Um, friends are fairly supportive. They’ve been coming swimming with me and yeah, yeah, my husband has also been coming swimming with me recently. Yeah, it’s been good.” (participant #9)*

#### THEME 4. Barriers to making change

A number of barriers to making change were identified, being intrinsic, extrinsic, and clinic-related.

- Sub-theme: Intrinsic

Intrinsic limitations for making change related to fatigue, medical problems making exercising more difficult, managing self-control and sensitivity discussing their weight.

*“there are days where I’m just so exhausted. I’m like, “Oh my god, I just want to skip it”, but I know that I really shouldn’t” (participant #7)*

*“I started going for walks like for about half an hour to 40 minutes a few times a week. But that stopped a little bit later, just due to pelvic pain and stuff like that. So I've just been doing shorter walks and getting in as much activity as I can.” (participant #3)*

*“making the changes can be really hard, definitely being pregnant. So when you want something, you want to eat that, it’s like, “Oh no, sorry, you can’t.” I’ve got to go home instead, and make a sandwich” (participant #8)*

A few women described difficulty talking about their weight during the consultation. They identified this as a sensitive issue, due to previous negative experiences.

*“I’ve been bullied, through school and stuff. I’m really self-conscious about my body…so, um, having to talk about it, it is um… It’s quite emotional.” (participant #8)*

Overall, most women had a practical attitude towards discussing their weight, and saw this as a necessary step in making change. Staff understand that weight is a sensitive issue and therefore approach GWG with care, working with women and taking a nuanced approach to conversations about weight, depending on the level of sensitivity.

*“I’ve always been a bit chubby…and I used to be very sensitive about it but I’m kind of like past that point now so it’s more… now that I’m pregnant, I wanted to be as healthy as I can possibly be…I knew that they were there to help basically so I just thought the best thing is to be as honest as possible rather than hide things.” (participant #14)*

- Sub-theme: Extrinsic

External barriers related to time, inclement weather, work, other children and finances.

*“my time constraints with my work schedule, so it wasn't anything that could really change… because I work shift work… my meal times would be different throughout the day. So sometimes because I would be at work I wouldn't have time to have a snack when I should have had a snack.” (participant #6)*

*“I was overloading (at university) and then I went away and did placement. So um that was stress and yeah, that was a barrier to making changes and then after that was finished, I kind of was able to make changes and I had the energy to make changes” (participant #9)*

*“Cost is always a factor, especially healthy food seems to be more expensive than junk food, which is really a pain. I’m like, “I love capsicums but they’re so expensive.”” (participant #9)*

Women with children acknowledged that caring for young children was an additional challenge, making exercise outside the home more difficult.

*“my 18 month old daughter really… with the exercise and that… but it's just with her, having her as well. Like it's been quite difficult to be able to get outside.” (participant #13)*

- Sub-theme: Clinic-related

Some women commented that their relationship with healthcare professionals made them feel somewhat uncomfortable initially. Some women acknowledged this may be due to their underlying sensitivity about their weight and felt that staff may have had preconceived ideas about women’s lifestyle.

*“I felt a bit judged…I felt that she just wanted me in, and just wanted me out. She didn’t smile.” (participant #8)*

*“the physician – she was a bit, I don’t know, distant, I guess. I found it a little bit difficult to kind of connect with her…I felt like I could make a decision if I wanted but um she was a bit resistant to what I was telling her to a certain extent, like her listening skills weren’t as good as they should be…it was probably just that I didn’t really click with her when I first met her but, you know, after that I kind of figured out how to kind of get the information that I wanted.” (participant #9)*

*“I know this is very much my perception of it, and it wasn't ever intended - but it was very much because you're obese, and you've got a high BMI, you're going to be higher risk for gestational diabetes, and blah, blah, blah. But they were saying all that before they knew that my diet and my exercise were actually quite good and I didn't need to change my diet… So I just - I kind of felt personally that they kind of assumed that you're going to have issues with trying to change your diet and all that sort of stuff.” (participant #6)*

Women commented on the waiting time, the fact that the clinic only ran on one particular weekday, and the parking expense as barriers.

*“it was only Wednesday afternoon from one o’clock onwards that it was available. It didn’t offer a lot of flexibility” (participant #2)*

*“pay for parking is a bit dear, next door… and just the waiting time sometimes. Like, not all the time, but the waiting time is a burden” (participant #1)*

#### THEME 5. Post-partum lifestyle and needs

- Sub-theme: Sustainable lifestyle changes

Women were able to identify lifestyle changes that could be sustained post-partum. Most felt that diet changes were more achievable than exercise, and recognised that intensity of changes may be reduced compared to pregnancy.

*“for sure…getting out of the house with a newborn is the best thing for you in terms of, you know, not getting depression and whatnot, which I experienced last time.” (participant #7)*

*“the food changes, the majority of the time I can keep up but it would probably be like less um – it won’t be as full on as it is now…I’ll be like a bit more relaxed about it definitely.” (participant #14)*

*“the main change was the eating habits and that sort of thing and they’ve pretty much stuck now so I’ll keep going the way I am.” (participant #5)*

Women without children were less confident in their ability to find time to exercise and cook healthy foods once their baby was born.

*“I hope that once the baby is born, I will stop being in pain and it’ll be easier to cook um and maintain like a good balance of meat and veggies and carbohydrates in my diet…hopefully I’ll be able to go back to walking the dogs.” (participant #9)*

*So I think um I would like to increase my exercise, again more once I'm not pregnant. The only difficulty will be that we'll be trying to do it with a newborn… it will just be once again like finding time to do - go food shopping, and meal preps, and all that sort of stuff, which I will, I will do. It's just now how will I do this with a baby as well. (participant #6)*

- Sub-theme: Support required to maintain lifestyle changes

Some, but not all women were interested in receiving support around diet and exercise post-partum. More women expressed interest in support in a face-to-face setting rather than via email. Some preferred group settings and others felt more comfortable with individual settings.

*“talking to other mothers who have children; um, you know, finding other strategies of you know, what they’re doing, how… how it’s worked for them, how it hasn’t worked for them” (participant #1)*

*“I think maybe diabetes wise, like I know I'm gestational, and I most likely may not have it after. But I think maybe ways to stop that from being something more permanent.” (participant #11)*

*“I think um especially exercising after you've had a baby, like knowing what you can do would be really beneficial for new mums, and making sure like little changes of interacting with your baby while you're making those healthy choices and stuff like that. Because you don't always know what you can and can't do physically after you've had the baby.” (participant #12)*

## Discussion

In this mixed-methods study evaluating pregnant women’s experiences of The Healthy Lifestyle in Pregnancy Project (HiPP), we identified patient perspective barriers and enablers for the implementation of an integrated healthy lifestyle intervention embedded in routine antenatal care for women with obesity. Overall, women have good risk perception and are motivated to make healthy lifestyle changes, but initially lack sufficient skills to implement them. Qualitative data identified themes of: service support enabled change; drivers of motivation to behaviour change; social support; barriers to making change and post-partum lifestyle and needs. Overall, qualitative and quantitative findings aligned. These learnings provide insight into important factors for improving the implementation model.

In early pregnancy, 70% of women reported gaining weight in the year prior. This is comparable to an Australian study of women in preconception, showing 54% had weight gain in the previous 12 months [38]. Here, the vast majority (80%) had attempted to lose weight, but had done so independently, with only 32% consulting a health professional. This concurs with findings from another preconception study, where few women had health checks prior to pregnancy to optimise their health and/or for weight management advice [39]. Generally, women had reasonable risk perception, and were able to recognise the target weight gain and risks related to excess weight gain, consistent with existing literature [40]. This may be partly explained by good background education levels and because women had completed their first midwife appointment (and possibly their first intervention session), where they would have received basic lifestyle information.

Of interest, women entered pregnancy with high expectations, all (100%) believed that they could manage healthy lifestyles and weight in pregnancy and almost all (97%) intended to take actions to prevent excess weight gain. However, when directly questioned about their confidence regarding eating and physical activity, confidence was lower at 74 and 67% respectively. This may be related to the style of question, with scaled question format more likely to reflect the person’s true feelings. Self-management strategy questions highlighted that women have good intentions for behaviour change but find implementation and relapse management difficult. This highlights that practising behaviour change is key to improving self-management and confidence, whilst reducing barriers. Additionally, women were more likely to be motivated to change and to keep track of their diet (55%) than exercise (41%). As both diet and exercise interventions offer benefits in pregnancy [9], interventions should aim to support enhancing both components.

Later in pregnancy, women reported strong satisfaction with the service provided, with 88% reporting satisfaction with information provided about lifestyle and 90% describing a good relationship with their healthcare provider. The reported satisfaction is higher than that reported in Australia in routine antenatal care [17]. This strong satisfaction may have contributed to improved self-management and improvement in diet (making time to prepare healthy meals, having food available for quick healthy meals, more likely to try new foods and recipes, and replacing snack foods with healthier alternatives). Additionally, women weighed themselves more regularly over the course of the intervention. Self-weighing has previously been shown to enhance intervention efficacy in the context of intervention support, but not in control groups without lifestyle support [41–43].

Quantitative data showed that women have healthy lifestyle intentions, but in some cases lack sufficient skills and confidence to implement them, emphasising that pregnancy is a ‘teachable moment’ [8]. Qualitative analysis explored how and why behaviour change was or was not made, and the strengths and weaknesses of the intervention. Key themes identified that the service clearly enabled change, with strong rapport between intervention staff and women. This confirms the overall high satisfaction women had with the service, with the majority of women having a good relationship with their health professional. Continuity of care with the same intervention staff promoted relationship building and trust, as described in other studies [26]. This social support is an important technique effective in lifestyle pregnancy interventions [44, 45]. Influence of family was an important theme in behaviour change, with a stronger support system facilitating positive change, with demonstrated reach to other close family members also shown. With women previously reported to be the main influencers of family lifestyle behaviours [46], this has significant public health implications, with potential for wider beneficial effects beyond individual improvements to health behaviours.

Exploratory studies have indicated that women desire clear, unambiguous and personalised strategies [13, 17, 18, 47, 48] for making lifestyle changes in pregnancy. Confidence is considered a key element for behaviour change during pregnancy according to the Theory of Planned behaviour [34, 49]. The challenging factor is how to enhance confidence and motivation to implement behaviour change. Here, women report that intervention staff largely provide this support, enabling success and a sense of achievement and improved confidence. When women saw they had healthy weight gain, this positively impacted their self-esteem, as previously described [50]. Women noted that dietary changes were easier to sustain as the pregnancy progressed, compared with physical activity, across both quantitative and qualitative data highlighting the need for effective, supportive strategies to target realistic and achievable physical activity goals. Quantitative analysis demonstrated improved self-management behaviours and in the qualitative component, a key theme was that the clinic enabled behaviour change, identifying a good fit of data integration.

Women identified a number of motivators for behaviour change, either intrinsic, due to concern regarding their baby’s well-being, or extrinsic such as being accountable to health professionals, reflecting international research [18, 26, 40, 51, 52]. Women also identified barriers to making change. Intrinsic (fatigue, medical problems, self-control) and extrinsic (time, inclement weather, work, other children and finances) barriers are universally recognised in this field, as women are challenged to balance everyday demands. Here, the interviews expanded insights of barriers gleaned from quantitative analysis. In contrast to many qualitative studies of health professionals’ experiences that describe reticence discussing obesity/gestational weight gain for fear of upsetting women [13–15], here very few women identified talking about their weight as a sensitive issue, and this may be due to intervention staff expertise in this area. Staff understand that weight is a sensitive issue and therefore approach GWG with care, working with women and taking a nuanced approach to conversations about weight, depending on the level of sensitivity. Reflecting on the relationship between healthcare providers and women, some described feeling judged in the initial stages, consistent with other studies [18]. Some women speculated this may be related to stigma around their weight and feeling vulnerable to negative attitudes that have been described previously [18, 26]. These feelings were expressed in the interview and not in the questionnaires, highlighting the benefit of qualitative analysis. Women wish to feel understood and treated with respect [53] and this feedback can expand learnings and be applied to improve the healthcare provider and recipient relationships.

There was variation in the anticipated sustainability of post-partum lifestyle changes between women with and without other children. Women without children were less confident in their ability to find time to exercise. Some women wanted postnatal support in varying formats. Engaging women postpartum is difficult and these factors need to be incorporated into future implementation models. Evidence is emerging that engagement in pregnancy and continuation post-partum is more successful than isolated post-partum approaches. With health benefits for mother and child demonstrated with healthy lifestyle in preconception, pregnancy and post-partum, a continuum approach would be ideal to support women at this high-risk period [54, 55].

Overall, this study demonstrates that women are gaining weight preconception and appear very motivated at the commencement of pregnancy to improve lifestyle, but lack of confidence hampers their success. Women want uncomplicated, clear advice. This intervention is designed to implement small, achievable changes that keep expectations realistic and remove the overwhelming feeling of having to change everything at once, by focussing on what is important to the woman at the time. Practising these techniques enhances self-management, problem solving and self-efficacy and changes are associated with weight gain prevention which in turn improves confidence in women. Facilitating factors are social support and rapport with intervention staff. Pregnancy is a time where increased support is needed by women and this intervention assisted in promoting this both within and outside of the intervention which is likely to be another factor associated with its success.

### Strengths and limitations

A strength of this research is the mixed-methods design, evaluating a pragmatic lifestyle intervention delivered embedded in routine maternity care, reflecting real world settings. By using quantitative and qualitative methods, we enriched our understanding of women’s experiences. In most aspects, results were strongly aligned, with coherence of quantitative and qualitative findings, with more in-depth insights from the thematic analysis. Additionally, the study included women from a low SES, diverse ethnic background catchment, increasing generalisability. The findings complement those of our health professionals’ perspectives [12]. Together, the studies have a role in informing implementation and scale of evidence-based, cost-effective antenatal lifestyle interventions. Possible limitations include the researcher’s prior clinical experience in the service that may have influenced interpretation of the participant’s response, however thematic analysis was completed by two independent researchers. Additionally, this experience is of a single clinical service in a larger health setting and will need to be generalised.

## Conclusions

Overall, healthy lifestyle was a high priority for pregnant women with obesity. Positive pregnancy care and lifestyle intervention experiences were reported, including satisfaction and being well-supported and involved. Prior to the intervention, women were able to identify strategies they could use to manage their lifestyle, but had less confidence to implement these changes, with confidence bolstered by the intervention. Ultimately, embedding an effective lifestyle intervention into routine care with dedicated trained health professionals enabled women to feel confident and empowered to make changes. Women identified weaknesses and strengths in their pregnancy care experiences and suggested ideas for improved service provision. Combining these findings with health professional perspectives will inform the scale-up of effective guideline recommended interventions in pregnancy more broadly.

## Supplementary Information

**Additional file 1.**

**Additional file 2.**

**Additional file 3.** Interview schedule for participants.

## Data Availability

Requests for de-identified data may be requested by written application to the corresponding author and will be considered on an individual basis.

## Abbreviations

BMI: Body mass index
GDM: Gestational Diabetes Mellitus
GWG: Gestational Weight Gain
HiPP: Healthy Lifestyle in Pregnancy Project
IOM: Institute of Medicine
RCT: Randomised controlled trial
