# Supplementary material for: Facilitators and barriers to behaviour change within a lifestyle program for women with obesity to prevent excess gestational weight gain: a mixed methods evaluation
Source: BMC Pregnancy Childbirth. 2021 Aug 18;21:569. doi: 10.1186/s12884-021-04034-7 (PMC8375116; doi:10.1186/s12884-021-04034-7)
Supplement: Supplementary file 1 — Additional file 1. [file 12884_2021_4034_MOESM1_ESM.pdf]

Questionnaire 1

PART 1

The following questions help us understand the women attending our services.

Please mark ONE response only to questions 1-5. There are no right or wrong answers.

1. **Do you believe your feedback about Dandenong Women's Antenatal Services can help improve the Service?**
   - ☐ Yes, definitely
   - ☐ Yes, moderately
   - ☐ Somewhat
   - ☐ No, not much
   - ☐ No, not at all

2. **We sent some pamphlets about pregnancy to you in the mail with your booking appointment. Were these pamphlets helpful?**
   - ☐ Yes, extremely
   - ☐ Yes, very
   - ☐ Somewhat
   - ☐ No, not much
   - ☐ No, not at all

3. **What is the highest level of schooling you have completed? (mark the highest grade)**
   - ☐ Year 10 or equivalent
   - ☐ Year 11 or equivalent
   - ☐ Year 12 or equivalent
   - ☐ Post school certificate/ diploma
   - ☐ Bachelor degree and above

4. **Do you currently work?**
   - ☐ Full time
   - ☐ Part time / casual
   - ☐ No paid work

5. **What is your average yearly income (before tax) that your household receives each year, including any financial support (eg. fortnightly benefits)?**
Household= salary of all income earners including yourself, partner and others

- ☐ $ 40,000 or less
- ☐ $41,000-64,000
- ☐ $65,000- 80,000
- ☐ more than $81,000

6. **Have you been diagnosed, or told by your doctor that you have any of the following? (cross all that apply)**

- ☐ Diabetes in pregnancy
- ☐ Type 1 diabetes
- ☐ Type 2 diabetes
- ☐ Heart disease
- ☐ High blood pressure
- ☐ Asthma
- ☐ Depression
- ☐ Cancer
- ☐ Polycystic ovarian syndrome (PCOS)
- ☐ Osteoarthritis
- ☐ I don't have any of these conditions

PART 2

1. **How often are you currently weighing yourself?**
☐ Daily ☐ Weekly ☐ Monthly ☐ Occasionally ☐ Never

2. **Have you gained weight in the past year (before you were pregnant)?**
☐ Yes ☐ No ☐ Unsure

3. **If yes, how much have you gained?**
- ☐ 1-2kg
- ☐ 3-4kg
- ☐ 5 kg
- ☐ 6-10kg
- ☐ greater than 10 kg

4. **Have you attempted to lose weight in the last 12 months?**
☐ Yes ☐ No

5. **In the past 12 months have you consulted a professional to help you manage your weight?**
☐ Yes ☐ No

If yes, who have you seen?

- ☐ Doctor
- ☐ Dietician
- ☐ Natural therapist
- ☐ Personal trainer
- ☐ Life coach
- ☐ Other (please describe______________________

6. In the past 12 months in order to help you have a healthy lifestyle, have you…? (cross all that apply)

- ☐ Increased vigorous exercise (eg. running, cycling)
- ☐ Cut down size of meal
- ☐ Cut down on snack foods and takeaway/ fats and sugar
- ☐ Vomited
- ☐ Skipped meals
- ☐ Increased smoking
- ☐ Attended a weight loss program (eg. Weight Watchers or Jenny Craig)
- ☐ Used meal replacement shakes or bars
- ☐ Had surgery (eg. gastric banding)
- ☐ Attended an structured exercise program (eg. personal trainer)
- ☐ Other (please describe) ____
- ☐ I have not made any changes

7. Before you were pregnant, how satisfied did you feel with

	Very dissatisfied	A little dissatisfied	Somewhat dissatisfied	Quite satisfied	Very satisfied
a. Your weight?	☐	☐	☐	☐	☐
b. Your body shape?	☐	☐	☐	☐	☐

PART 3

1. How much weight gain during your pregnancy do you believe is best for your health and the health of your baby? (please select one only)

- ☐ No weight gain
- ☐ 0-5 kg
- ☐ 5-9 kg
- ☐ 9-15kg
- ☐ 15-20 kg
- ☐ 20kg or above

2. **I believe that increased weight gain in pregnancy is associated with: (please select all that apply)**
   - ☐ More nutrients for the baby
   - ☐ Big babies (macrosomia)
   - ☐ Early delivery (preterm labour)
   - ☐ Need for induction of labour
   - ☐ Diabetes in pregnancy (gestational diabetes)
   - ☐ High blood pressure in pregnancy
   - ☐ Baby kicking more
   - ☐ Baby not feeding well after delivery
   - ☐ Mother developing diabetes in the future
   - ☐ Your child becoming overweight as a child or an adult
   - ☐ Baby developing asthma
   - ☐ None of the above

Please answer the following statements according to how you were thinking when you first became aware that you were pregnant

1. **I think it is important to have a healthy lifestyle during pregnancy**

Strongly agree	Agree	Somewhat agree	Disagree	Strongly disagree
☐	☐	☐	☐	☐

2. **I thought I was at risk of gaining too much weight in pregnancy**

Strongly agree	Agree	Somewhat agree	Disagree	Strongly disagree
☐	☐	☐	☐	☐

3. **I believe I can manage to have a healthy lifestyle and healthy weight gain in pregnancy**

Strongly agree	Agree	Somewhat agree	Disagree	Strongly disagree
☐	☐	☐	☐	☐

4. **I plan to take action to prevent too much weight gain**

Strongly agree	Agree	Somewhat agree	Disagree	Strongly disagree
☐	☐	☐	☐	☐

PART 4

Complete every question by drawing a circle around the number that describes how you feel.

1. **How <u>important</u> is it to you to make healthy lifestyle changes during your pregnancy regarding your eating?**

Not at all -- Completely

1 2 3 4 5 6 7 8 9 10

2. **How <u>important</u> is it to make healthy lifestyle changes during your pregnancy regarding your physical activity?**

Not at all -- Completely

1 2 3 4 5 6 7 8 9 10

3. **How <u>ready</u> are you to make healthy lifestyle changes during your pregnancy regarding your eating?**

Not at all -- Completely

1 2 3 4 5 6 7 8 9 10

4. **How <u>ready</u> are you to make healthy lifestyle changes during your pregnancy regarding your physical activity?**

Not at all -- Completely

1 2 3 4 5 6 7 8 9 10

5. **How <u>confident</u> are you to make healthy lifestyle changes during your pregnancy regarding your eating?**

Not at all -- Completely

1 2 3 4 5 6 7 8 9 10

6. **How <u>confident</u> are you to make healthy lifestyle changes during your pregnancy regarding your physical activity?**

Not at all -- Completely

1 2 3 4 5 6 7 8 9 10

PART 5

We are interested in your feelings over the last 12 months.

Complete every question by crossing one box like this: x

	Never	Occasionally	Often	Very often	Always
**1. I think about the benefits I will get from being physically active**	☐	☐	☐	☐	☐
**2. I try to think more about the benefits of physical activity and less the hassles of being active**	☐	☐	☐	☐	☐
**3. I make backup plans to be sure I get enough physical activity**	☐	☐	☐	☐	☐
**4. When I get off track with my physical activity I find ways to get back on track**	☐	☐	☐	☐	☐
**5. I ask friends and family to walk with me to help me stay active**	☐	☐	☐	☐	☐
**6. I can stick to my plans to be active each week**	☐	☐	☐	☐	☐
**7. When I set goals I choose activities that I enjoy**	☐	☐	☐	☐	☐
**8. I know when I should do more activity**	☐	☐	☐	☐	☐
**9. I plan ahead of time to be active**	☐	☐	☐	☐	☐
**10. I look for information about nutrition and healthy eating from books, magazine, internet etc**	☐	☐	☐	☐	☐
**11. I read articles about the benefits of being active from magazines, books or the internet**	☐	☐	☐	☐	☐
**12. I seek information about my weight from my GP**	☐	☐	☐	☐	☐

**13. I keep track of how much physical activity I do each week**	☐	☐	☐	☐	☐
**14. I do things to make walking or other activity more enjoyable**	☐	☐	☐	☐	☐
**15. I watch my weight**	☐	☐	☐	☐	☐
**16. I watch what I eat**	☐	☐	☐	☐	☐
**17. I keep track of what I eat and know how much I should eat**	☐	☐	☐	☐	☐
**18. I can stop myself overeating**	☐	☐	☐	☐	☐
**19. I say positive things to myself about eating healthy food**	☐	☐	☐	☐	☐
**20. If I don't eat healthy food I think about ways to do better next time**	☐	☐	☐	☐	☐
**21. I make plans to change my diet/ drinking habits**	☐	☐	☐	☐	☐
22. **I weigh myself regularly**	☐	☐	☐	☐	☐
**23. I read labels to help me choose healthy food**	☐	☐	☐	☐	☐
**24. I make sure I have time to prepare healthy meals**	☐	☐	☐	☐	☐
**25. I have food available for quick healthy meals**	☐	☐	☐	☐	☐
**26. I try new foods and recipes to make healthy food enjoyable**	☐	☐	☐	☐	☐
27. **I eat healthy food**	☐	☐	☐	☐	☐
**28. I replace snack foods with healthier alternatives**	☐	☐	☐	☐	☐
**29. I decide what to eat at the last minute**	☐	☐	☐	☐	☐

Thank you very much for completing the questionnaire. We appreciate your time.

Please take a moment to check and see that you have completed all the questions.
