# Supplementary material for: Facilitators and barriers to behaviour change within a lifestyle program for women with obesity to prevent excess gestational weight gain: a mixed methods evaluation
Source: BMC Pregnancy Childbirth. 2021 Aug 18;21:569. doi: 10.1186/s12884-021-04034-7 (PMC8375116; doi:10.1186/s12884-021-04034-7)
Supplement: Supplementary file 2 — Additional file 2. [file 12884_2021_4034_MOESM2_ESM.pdf]

Questionnaire 2

PART 1

Please mark ONE response to each of these questions. There are no right or wrong answers.

1. **During your pregnancy care, how satisfied were you with the information that you obtained about a healthy lifestyle (including dietary changes and exercise)?**

☐ Very satisfied
☐ Satisfied
☐ Neither satisfied nor dissatisfied
☐ Dissatisfied
☐ Very dissatisfied
☐ This information was not mentioned

2. **Has your antenatal care team been helpful in assisting and supporting you to be more physically active?**

☐ Yes, extremely
☐ Yes, very
☐ Somewhat
☐ No, not much
☐ No, not at all

3. **Has your antenatal care team been helpful in assisting and supporting you to have healthy eating?**

☐ Yes, extremely
☐ Yes, very
☐ Somewhat
☐ No, not much
☐ No, not at all

4. **Did you feel you had a good relationship with your health professional team?**

☐ Yes, extremely
☐ Yes, very
☐ Somewhat
☐ No, not much
☐ No, not at all

5. **Were you given enough time to ask questions and discuss your pregnancy health during your appointments?**

☐ Yes, definitely
☐ Yes, very
☐ Somewhat
☐ No, not much
☐ No, not at all

6. **Did you feel involved in decision making about your pregnancy care?**

☐ Yes, definitely
☐ Yes, very
☐ Somewhat
☐ No, not much
☐ No, not at all

PART 2

1. **I took definite actions during my pregnancy to have a healthy lifestyle and reduce my risk of gaining too much weight**

☐ Yes, definitely
☐ Yes, mostly
☐ Somewhat
☐ No, not much
☐ No, not at all

<u>If you answered 'yes', please complete Q 2 to 5. If you answered 'no' please go to question 6.</u>

2. **What would you consider to be the main changes you made?**
(Please mark all the ones that apply)

☐ I eat more fruit and vegetables
☐ I drink more water
☐ I eat more low fat dairy products (e.g. milk, cheese and yoghurt)
☐ I have less fruit juice, cordial and soft drink
☐ I have less snack foods (e.g. chocolate, chips etc)
☐ I have less takeaway and convenience foods

- ☐ I have increased the <u>number</u> of times I exercise each week
- ☐ I have increased the <u>time</u> I spend on each exercise session
- ☐ I have increased the physical <u>intensity</u> of exercise sessions
- ☐ I make more time for relaxation
- ☐ Other ___________________________________

3. I have worked <u>on maintaining the lifestyle changes</u> I have made to <u>my diet</u>

- ☐ Yes, definitely
- ☐ Yes, very
- ☐ Somewhat
- ☐ No, not much
- ☐ No, not at all

4. I have worked <u>on maintaining the lifestyle changes</u> I have made to <u>my physical activity</u>

- ☐ Yes, definitely
- ☐ Yes, very
- ☐ Somewhat
- ☐ No, not much
- ☐ No, not at all

5. How confident are you that you can maintain any lifestyle changes that you have made after you deliver your baby?

- ☐ Very confident
- ☐ Confident
- ☐ Partly confident
- ☐ A little confident
- ☐ Not confident at all

6. **What would you consider to be the main barriers to making any lifestyle changes to your diet and physical activity?**

- ☐ Lack of time
- ☐ Fatigue/tiredness
- ☐ Taking care of other children
- ☐ Family/spousal influences
- ☐ Change in circumstance (i.e. moving house, going overseas)
- ☐ Change in work situation
- ☐ Illness (yourself or another family member)
- ☐ Too much effort
- ☐ No motivation/ feeling lazy
- ☐ Not confident in making changes
- ☐ Lack of support from family/friends
- ☐ Bad weather
- ☐ Not important to me
- ☐ Other ____

7. **How often are you currently weighing yourself?**

☐ Daily ☐ Weekly ☐ Monthly ☐ Occasionally ☐ Never

PART 3

We are interested in your feelings during this pregnancy.

Complete every question by crossing one box like this: ☒

	Never	Occasionally	Often	Very often	Always
**1. I think about the benefits I will get from being physically active**	☐	☐	☐	☐	☐
**2. I try to think more about the benefits of physical activity and less the hassles of being active**	☐	☐	☐	☐	☐
**3. I make backup plans to be sure I get enough physical activity**	☐	☐	☐	☐	☐
**4. When I get off track with my physical activity I find ways to get back on track**	☐	☐	☐	☐	☐
**5. I ask friends and family to walk with me to help me stay active**	☐	☐	☐	☐	☐
**6. I can stick to my plans to be active each week**	☐	☐	☐	☐	☐
**7. When I set goals I choose activities that I enjoy**	☐	☐	☐	☐	☐
**8. I know when I should do more activity**	☐	☐	☐	☐	☐
**9. I plan ahead of time to be active**	☐	☐	☐	☐	☐
**10. I look for information about nutrition and healthy eating from books, magazine, internet etc**	☐	☐	☐	☐	☐
**11. I read articles about the benefits of being active from magazines, books or the internet**	☐	☐	☐	☐	☐
**12. I seek information about my weight from my GP**	☐	☐	☐	☐	☐

**13. I keep track of how much physical activity I do each week**	◻	◻	◻	◻	◻
**14. I do things to make walking or other activity more enjoyable**	◻	◻	◻	◻	◻
**15. I watch my weight**	◻	◻	◻	◻	◻
**16. I watch what I eat**	◻	◻	◻	◻	◻
**17. I keep track of what I eat and know how much I should eat**	◻	◻	◻	◻	◻
**18. I can stop myself overeating**	◻	◻	◻	◻	◻
**19. I say positive things to myself about eating healthy food**	◻	◻	◻	◻	◻
**20. If I don't eat healthy food I think about ways to do better next time**	◻	◻	◻	◻	◻
**21. I make plans to change my diet/ drinking habits**	◻	◻	◻	◻	◻
22. **I weigh myself regularly**	◻	◻	◻	◻	◻
**23. I read labels to help me choose healthy food**	◻	◻	◻	◻	◻
**24. I make sure I have time to prepare healthy meals**	◻	◻	◻	◻	◻
**25. I have food available for quick healthy meals**	◻	◻	◻	◻	◻
**26. I try new foods and recipes to make healthy food enjoyable**	◻	◻	◻	◻	◻
27. **I eat healthy food**	◻	◻	◻	◻	◻
**28. I replace snack foods with healthier alternatives**	◻	◻	◻	◻	◻
**29. I decide what to eat at the last minute**	◻	◻	◻	◻	◻

Thank you very much for completing the questionnaire. We appreciate your time.

Please take a moment to check and see that you have completed all the questions.
