# Supplementary material for: Facilitators and barriers to behaviour change within a lifestyle program for women with obesity to prevent excess gestational weight gain: a mixed methods evaluation
Source: BMC Pregnancy Childbirth. 2021 Aug 18;21:569. doi: 10.1186/s12884-021-04034-7 (PMC8375116; doi:10.1186/s12884-021-04034-7)
Supplement: Supplementary file 3 — Additional file 3. Interview schedule for participants. [file 12884_2021_4034_MOESM3_ESM.docx]

**Additional file 3**

**Interview schedule for participants**

- **Explore maternity service engagement**

We would like to get your opinion on what you liked/disliked about attending the maternity clinic, in particular your experience with the health coach and endocrinologist.

1. What was positive?

Prompt: Did you feel the aims of the clinic were explained to you?

What was your relationship like with the obstetrician/midwife/endocrinologist/health coach?

How were you involved in decision making?

Did you feel like you had the opportunity to ask questions?

1. What was negative?

Prompt: Was the time of the clinic (afternoon) an issue?

We know you may have been seeing lots of people on the one day, was waiting time an issue?

- **Behaviour change**

1. What advice were you given about having a healthy pregnancy?

Prompt: Tell me about the advice the health coach/obstetrician/endo/midwife gave you?

What advice were you given about diet/food/exercise/weighing yourself?

1. What did you feel were the main goals of the lifestyle sessions?

What did you find most beneficial?

Was the level of support provided adequate to enable you to achieve a healthier lifestyle in pregnancy?

Following the sessions were you ready to change?

If you did make any changes, can you tell me what they were?

1. What helped you make these changes?

Prompt: setting your own goals/ weighing yourself/ practical advice eg. food substitution/ knowing you were coming back for each review/ breaking down larger changes into smaller sustainable changes

1. Thinking about the changes you made, did it make you feel more confident in yourself?
2. Some women found it difficult to make changes. Were there any barriers that prevented you from fulfilling lifestyle goals?

Prompt: Family responsibilities/work responsibilities (too busy)/ cost (too expensive)/easier to wait until the pregnancy is complete and then starting afresh with lifestyle changes

1. Can you think of anything else the maternity clinic could have provided differently to make this easier for you?
2. If you have made changes, have you found your family/friends are supportive? In what way?
3. Have your changes had any impact on your family and friends?

Prompt: Are you buying/cooking different food?

Are you exercising as a family?

1. Tell me about anything you found challenging or difficult in the clinic

Prompt: Talking about your weight or eating habits

Can you describe this experience? Was it supportive?

1. Thinking about the changes you have made, do you feel they are sustainable to you? (eg. can you continue any of these changes after your baby is born?) Which ones do you think you are most likely to keep up?

Prompt: Diet/exercise/weighing/sleep

1. Is there any support you would like to receive in the first 6 months after having a baby?

Prompt: about staying healthy, eating well and exercising?

Would you prefer to receive this to be face-to-face (appointment) or written (mail/email)?

If face-to-face, would you prefer this individually or in a group session with other new mums? (ie. part of maternal child health experience)

Is there anything more you would like to add?

**Demographic questions** (only for women who have not completed questionnaire 1)

**What is the highest level of schooling you have completed? (mark the highest grade)**

Year 10 or equivalent

Year 11 or equivalent

Year 12 or equivalent

Post school certificate/ diploma

Bachelor degree and above

**Do you currently work?**

Full time

Part time / casual

No paid work

**What is your average yearly income (before tax) that your household receives each year, including any financial support (eg. fortnightly benefits)?**

*Household= salary of all income earners including yourself, partner and others*

$ 40,000 or less

$41,000-64,000

$65,000- 80,000

more than $81,000

**Have you been diagnosed, or told by your doctor that you have any of the following? (cross all that apply)**

Diabetes in pregnancy

Type 1 diabetes

Type 2 diabetes

Heart disease

High blood pressure

Asthma

Depression

Cancer

Polycystic ovarian syndrome (PCOS)

Osteoarthritis

I don’t have any of these conditions
